# Consensus statements and clinical recommendations on treatment indications, surgical procedures, prosthetic protocols and complications following All-On-4 standard treatment. 9th Mozo-Grau Ticare Conference in Quintanilla, Spain

**DOI:** 10.4317/jced.53759

**Published:** 2017-05-01

**Authors:** Miguel Penarrocha-Diago, María Penarrocha-Diago, Regino Zaragozí-Alonso, David Soto-Penaloza, Meeting on behalf of the Ticare Consensus

**Affiliations:** 1 MD, DMD, PhD, Professor and Chairman of Oral Surgery, Stomatology Department, Faculty of Medicine and Dentistry, University of Valencia, Spain; 2 MD, DDS, PhD, Assistant Professor of Oral Surgery, Stomatology Department, Faculty of Medicine and Dentistry, University of Valencia, Spain; 3 DDS, Dentist, Department of Stomatology, Faculty of Medicine and Dentistry, University of Valencia, Spain; 4 DDS, MSc, Collaborating Lecturer, Master in Oral Surgery and Implant Dentistry, Department of Stomatology, Faculty of Medicine and Dentistry, University of Valencia, Spain; 5 Juan Antonio Blaya-Tárraga, University of Valencia, Spain; Abel García-García, University of Santiago de Compostela, A Coruña, Spain; Agustin Ripoll, Specialist Technician in Dental Prosthodontics, Valencia, Spain; Alberto Fernández-Ayora, Private practice, Almería, Spain; Alberto Fernandez-Sanchez, Private practice, Almería, Spain; Ana Orozco-Varo, University of Seville, Spain; Antonio Juan Flichy-Fernández, University of Valencia, Spain; Arturo Sánchez-Pérez, University of Murcia, Spain; Carlos Bonilla-Mejías, University of Seville, Spain; Carlos Larrucea-Verdugo, University of Talca, Chile; Carlos Sáenz-Ramírez, University of Seville, Spain; Daniel Robles-Cantero, CEPUME, University of Alcalá de Henares, Madrid, Spain; Florencio Monje-Gil, University of Badajoz, Spain; Alberto González-Garcia, University of Seville, Spain; Angels Pujol-García, International University of Catalonia, Barcelona, Spain; Javier Ortolá-Dinnbier, Specialist Technician in Dental Prosthodontics, Valencia, Spain; Javier Valladares-Relaño, Specialist Technician in Dental Prosthodontics, Salamanca, Spain; Luis Miguel Vera-Fernández, Specialist Technician in Dental Prosthodontics, Seville, Spain; María Isabel González-Martin, University of Seville, Spain; Pablo Domínguez-Cardoso, University of Seville, Spain; Raúl Fernández-Encinas, Private practice, Valladolid, Spain

## Abstract

**Objectives:**

The present consensus report critically evaluates the scientific evidence based on a comprehensive systematic review of the All-On-4 treatment concept, focusing primarily on the treatment indications, surgical procedures and prosthetic protocols, and secondarily on the mechanical and biological complications involved.

**Material and Methods:**

A systematic review was made in advance of the meeting. Consensus statements, treatment guidelines and recommendations for future research were based on within-group as well as plenary debates and discussions of the systematic review.

**Results:**

The main indication of All-On-4 standard care is an atrophic maxilla or mandible, with or without remnant hopeless tooth. in ASA I or II patients. This surgical-prosthetic protocol seems efficient, safe and effective in the case of Cawood & Howell class IV, V and VI. It is necessary for the implant to have had an insertion torque of over 35 Ncm for immediate loading. The provisional prosthesis should provide rigidity, being non-flexible in order to avoid micro-movements, and should be strong enough to not fracture. Balanced occlusion without interferences is required, ensuring very gentle dynamic movements. The design of the definitive prosthesis must be cleanable and biomechanically adjusted to the implant position and individual characteristics of each patient. A non-concave acrylic base resting over soft tissue is recommended, facilitating hygiene. Regarding occlusion, a group guide should be made, taking into account whether the antagonist is not a removable complete denture. In that case, bi-balanced occlusion should be assessed. Prosthetic complications occur as a result of fractures of the provisional acrylic prostheses. These problems in turn can be resolved by repair through relining or fixing. The most frequent biological complication is the loss of at least one implant, while the second most frequent complication is the development of peri-implantitis and mucositis.

**Conclusions:**

In the treatment of atrophy for full-arch implant supported restorations it is considered that four implants suffice for immediate loading and the final prosthesis, even when there is available bone between the mental foramina or maxillary sinuses. The weakness of the quality of the available evidence indicates that further studies are needed, involving an appropriate design and with adequate follow-up in All-On-4 standard care to confirm the present results mainly in relation to survival rates and complications.

** Key words:**Atrophic jaw, All-on-4, immediate implant loading, edentulous mandible, edentulous maxilla, tilted implant, implant failure, dental implants.

## Introduction

There is persistent controversy on the ideal rehabilitation of edentulous patients. Immediate loading procedures for edentulous jaws have become widely popular among clinicians (1,2), with great acceptance on the part of the patients (3).

The challenge today is not to prove functionality but rather to develop simple and cost-effective protocols, ensuring the wellbeing of patients. The present report summarizes the statements and clinical recommendations referred to the All-On-4 treatment concept, based on a consensus agreement among the participants at the Ninth Ticare Conference held in Quintanilla (Valladolid, Spain).

The All-on-4 treatment concept involves the placement of four implants in the anterior part of the maxilla or in the inter-foramina space of the jaw - two mesial axial implants and two angled implants in the distal position - to support a fixed immediate loading full-arch prosthesis. This treatment strategy offers promising results over short and middle term, and proves highly successful in terms of survival rates, as documented by the scientific literature (4).

However, such treatment constitutes a special challenge in every-day clinical practice, with doubts remaining regarding the specific treatment indications, surgical procedures and prosthetic protocols following the All-On-4 procedure. In this regard, the effects of the treatment strategies in terms of patient satisfaction, and the treatment complications (both technical and biological) remain to be firmly established.

The present consensus report critically evaluates the scientific evidence based on a comprehensive systematic review of the All-On-4 treatment concept, focusing primarily on the treatment indications, surgical procedures and prosthetic protocols (loading time, prosthesis material, abutment, type of fixation, occlusal control), and secondarily on the mechanical and biological complications such as implant failure, mucositis, peri-implantitis or prosthesis fracture, abutment fracture, screw fracture or losses.

Furthermore, an analysis is made of the clinical outcomes of this treatment concept over different time spans of at least three years after immediate loading. The discussion led to the development of statements and recommendations determined through group consensus based on the findings of the systematic review.

-Focus question

 “What are the most frequent clinical indications, surgical procedures, prosthetic protocols and complications in edentulous patients or individuals with severely resorbed jaws receiving dental implants for immediate full-arch implant supported restorations following the All-On-4 concept in the mandible or maxilla?”.

-Consensus statements

Depicted by each comprehensive and complete objective.

-Clinical indications

The main indication of All-On-4 standard care is an atrophic maxilla or mandible with or without remnant hopeless tooth in ASA I or II patients. This surgical-prosthetic protocol seems efficient, safe and effective in Cawood & Howell class IV, V and VI. The procedure requires minimum dimensions of the alveolar process in the maxilla between the mesial wall of the maxillary sinuses and between the emergence of the mental nerves in the jaw, in order to allow placement of the four implants.

Another indication refers to patients reluctant to undergo bone regenerative procedures such as sinus lift, bone grafting or transposition of the dental nerve. Moreover, the protocol is indicated in accordance to the prosthetic design involved – the patient being required to have 12 mm of height in the prosthetic space, without the need for a buccal flank to give lip support.

For immediate loading, the implants must have had an insertion torque of over 35 Ncm.

-Surgical procedures

Local anesthesia using the infiltration technique is advised, complemented with oral or intravenous sedation.

The preparation of a surgical-radiological splint is suggested, based on a waxed diagnostic model, or adopting a computer-designed surgical splint, when a flapless approach is used.

When a flapless technique is not performed, a crestal incision from the first molar to the contralateral molar is made, with or without distal discharge. In the mandible, distal discharge is made after emergence of the mental nerve in order to avoid injuries. After flap reflection and detection of the mental foramina in jaw, the length of the mental nerve loop as well as the shape of bone are assessed in order to determine the ideal angulation for the posterior implants. Before implant placement, all compromised teeth must be extracted, and the sockets are to be carefully debrided.

If a window is made in the maxillary sinus, introducing a periodontal probe allows us to locate the medial wall of the sinus, thereby guiding distal implant placement. Moreover, by using current diagnostic tools such as cone-beam computed tomography, with the surgical splint, it is possible to locate the medial limit of the maxillary sinus, avoiding perforation of the Schneiderian membrane.

Alveolar ridge regularization allows standard diameter implants to be placed in the correct position. Another advantage is that dentogingival prostheses can be placed, thereby enhancing aesthetics, since the gingiva-prostheses interface is located apical from the smile line.

The angulation of the distal implants should be between 30° and 45°, depending on the situation and anatomical location. Use is made of implants with a diameter of 4 mm and a minimum length of 10 mm and 11.5 mm, axial and distal respectively. Moreover, in the case of the latter implant, it is advisable to place the greatest length allowed by planning. A measuring tool is recommended for assessing primary stability.

-Prosthetic protocols

•Abutment type and prosthetic screw tightness 

Abutments with an inclination of between 17° and 30° are advised in order to compensate the lack of parallelism between im-plants. Regarding prosthetic screw tightening, forces of around 10-20 Ncm are suggested.

•Provisional prosthesis material

The provisional prosthesis should provide rigidity, being non-flexible in order to avoid micro-movements that impede the implant osseointegration process, and should be strong enough to not fracture.

High-density acrylic resin materials are to be preferred, depending on the prosthetic-space dimension, as they can ensure rigidity and prevent fractures. Depending on the patient characteristics, and particularly in cases with little space, the prostheses can be reinforced with a metal frame.

We can place 10 or 12 teeth without cantilever, depending on whether the implants emerge up to the second premolar or the first molar.

•Immediate loading and occlusion

Immediate loading is to be performed during the first 24 hours and until one week after surgery. The provisional prosthesis must remain in place at least 6 months, allowing stabilization of the soft tissue, and should be resistant to fractures - thereby avoiding the need for prostheses removal for repair.

During immediate loading, balanced occlusion without interferences is required, ensuring very gentle dynamic movements, since immediate loading seeks rigidity of the prosthesis, and it is essential to avoid disrupting the osteointegration process.

•Definitive prostheses material and occlusion

The definitive prosthesis may be made using metal-ceramic/acrylic resin materials, reinforced with metal frameworks; the denture extension consists mainly of up to 12 teeth. Prosthetic manufacturing using new technologies such as CAD-CAM and other new materials not validated to date can be performed as a way to explore and change the classic materials used for many years.

The prosthesis structure must be adapted to the coating or veneering material. It is advisable to work with the option of easy repair in situations of cracked prostheses. The design of the definitive prosthesis must be cleanable and biomechanically adjusted to the implant position and individual characteristics of each patient, e.g., age, sex, functional and parafunctional habits, muscle tone, antagonist, etc.

A non-concave acrylic base resting above the soft tissue is advised, facilitating hygiene, and avoiding plaque-induced complications. Regarding occlusion, a group guide should be made, taking into account whether the antagonist is not a removable complete denture. In that case, bi-balanced occlusion should be assessed.

•Prosthetic settlement assessment

Prosthetic settlement must be verified through panoramic and periapical radiographs, using the parallel projection technique to assess and guide fitting of the prostheses and abutments.

-Complications

•Mechanical

Prosthetic complications occur as a result of fractures of the provisional acrylic prostheses. These problems can be solved by repairing through relining or fixing, adjusting the occlusion, and using an occlusal splint. The detachment of an element of the definitive prosthesis is the most frequent problem. However, this does not affect the survival rate of either implants or prostheses.

•Biological

The most frequent biological complication is the loss of at least one implant, while the second most frequent complication is the development of peri-implantitis and mucositis.

-Treatment guidelines

•Indications 

Patients are required to have a minimum alveolar process dimension allowing the placement of four implants in the pre-maxilla zone, and without needing a buccal flank to hold up the lip. A torque of at least 35 Ncm is required to perform immediate loading.

•Prosthetic protocol

▫Abutment type and prosthetic screw tightness 

The abutments should be tight, applying the forces indicated by the manufacturer, because screw losses or fractures of both the provisional and prosthetic fitting can result from mishandling, misfit and occlusal forces.

▫Immediate loading and occlusion

Immediate loading is to be performed during the first 24 hours and until one week after surgery. The provisional prosthesis must remain in place at least 6 months, allowing stabilization of the soft tissue, and should be resistant to fractures - thereby avoiding the need for prostheses removal for repair or relining.

We therefore need to ensure an adequate design, with appropriate material and references regarding the prosthetic space - balanced occlusion without interferences being mandatory.

Fracture of the provisional acrylic prosthesis and the detachment of an element of the definitive prosthesis are common problems in the All-On-4 standard concept. Consequently, it is very important to minimize the risk through adequate occlusal control with group function, avoiding the canine guide in definitive prostheses, and assessing the prosthetic space to establish the best design according to the individual characteristics of the patient, with due consideration of the antagonist.

▫Prosthetic settlement assessment 

Panoramic and periapical radiograph should be used to assess fitting between the implant connection and the prosthetic parts during maintenance recall, thereby contributing to avoid future mechanical complications.

-Biological complications 

Because the high prevalence of peri-implant diseases such as mucositis and peri-implantitis, in this sense, the recommendation is to consider that it is utmost important define the disease with a pre-established bone loss threshold, assessing systemic and local risk factors, as well as, implementing a customized maintenance protocol for implants, according individualized features by each patient.

## General Statements

-General clinical recommendations

The current trend is to place fewer implants than only few years ago. Starting from protocols comprising 8 and 6 implants, the numbers has since been maintained or reduced.

-General recommendations for future research

Definition should be made of the degree of atrophy and the type of patient in which the all-on-four standard is indicated.

Prospective clinical trials should be designed to compare four implants versus the classic number of 6 and 8, in order to assess the advantages and disadvantages in terms of the incidence of complications and the survival rates associated to the different procedures, ensuring a sufficient sample size and adequate follow-up.

New types of currently available materials should be evaluated, following the All-On-4 concept.

Dental implants should be designed and manufactured for placement in an angulated distal position, with a modified angled connection emulating zygomatic implants, in an attempt to minimize microleakage generated by gaps at the implant-abutment interface.
